# Prognostic Value of Postoperative Circulating Tumor DNA in Patients With Early- and Intermediate-Stage Hepatocellular Carcinoma

**DOI:** 10.3389/fonc.2022.834992

**Published:** 2022-03-04

**Authors:** Ke Ye, Qinqiao Fan, Mingming Yuan, Dong Wang, Liang Xiao, Guo Long, Rongrong Chen, Tongdi Fang, Zengbo Li, Ledu Zhou

**Affiliations:** ^1^ Department of General Surgery, Xiangya Hospital, Central South University, Changsha, China; ^2^ Department of Hepatobiliary Tumour Surgery, Chenzhou No.1 People’s Hospital, Chenzhou, China; ^3^ Department of Medicine, Geneplus-Beijing, Beijing, China; ^4^ Department of Liver Disease Center, The Affiliated Hospital of Qingdao University, Qingdao, China

**Keywords:** circulating tumor DNA, liver cancer, alpha-fetoprotein, next-generation sequencing, postoperative recurrence

## Abstract

Majority of patients with resected early- and intermediate-stage liver cancer will experience postoperative recurrence. This study aimed to investigate the application of ctDNA sequencing in the postoperative period of hepatocellular carcinoma. A total of 96 patients with liver cancer were enrolled in this study. Postoperative peripheral blood samples were collected from all patients after surgery and analyzed using hybridization capture-based next-generation sequencing. Identification of at least one somatic mutation in the peripheral blood was defined as ctDNA+. Five genetic features in tumor tissues were associated with disease-free survival (DFS) using Lasso-Cox model. The area under the receiver operating characteristic curve was 0.813 and 0.882 in training and validation cohorts, respectively. The recurrence rate in ctDNA+ and ctDNA- groups was 60.9% and 27.8%, respectively. Multivariate Cox regression analysis showed that the postoperative ctDNA was an independent prognostic predictor of DFS (HR [hazard ratio]: 6.074, 95% Cl [confidence interval]: 2.648-13.929, P<0.001) and overall survival (OS) (HR: 4.829, 95% CI: 1.508-15.466, P=0.008). Combined ctDNA with AFP improved prediction performance. The median DFS was 2.0, and 8.0 months in ctDNA+/AFP-H and ctDNA+/AFP-L groups, respectively; while ctDNA-/AFP-H and ctDNA-/AFP-L groups had not reached the median time statistically (Log-rank test, *P* < 0.0001). Furthermore, ctDNA- patients had better prognosis than ctDNA+ patients irrespective of tumor stage. Postoperative ctDNA sequencing has great prognostic value in patients with liver cancer. Patients with positive ctDNA should receive more intensive disease monitoring and more aggressive treatment strategies to improve the survival time.

## Background

The incidence and mortality of hepatocellular carcinoma (HCC) are among the top five in all tumors in China (1). Evidence suggests that surgical resection, liver transplantation and other curative therapies can significantly improve the survival for those HCC patients with tumor diagnosed at early stage (2). However, the overall survival of HCC patients remains unsatisfactory due to frequent tumor recurrence and metastasis after curative treatments (3). Since the recurrence rate of HCC within 5 years following liver resection has been reported to exceed 70% (4), it is of vital importance to effectively identify those patients who were at a high risk of recurrence after surgery.

Various prognostic tools which interpret surrogate clinicopathologic features such as liver cirrhosis, tumor size, vascular invasion and serological markers have been proposed to predict HCC tumor recurrence with varying degree of reliability (5, 6). Although these prognostic markers are related to tumor recurrence and patient survival, it is difficult to accurately identify those patients who are at a high risk of recurrence immediately after surgery. Furthermore, there is a lack of effective markers to identify earlier staged patients who require more aggressive adjuvant therapy.

Circulating tumor DNA (ctDNA) had been reported as a potent biomarker to reflect tumor load and treatment efficacy in varieties of cancers irrespective of tumor stage (7–10). Previous study has shown that among patients with colorectal cancer, the recurrence rate of ctDNA-positive patients was 77% and ctDNA-negative patients were 0% during the median follow-up time (11). In patients with liver cancer, ctDNA has been proved to be associated with clinical characteristics and clinicopathologic parameters, and could be used as a non-invasive biomarker to monitor tumor progression in real-time (12–14). However, at present, alpha-fetoprotein (AFP) and computed tomography (CT) are routinely used methods for postoperative monitoring (15, 16).

In this study, we aimed to verify the clinical value of postoperative ctDNA in patients with early- or intermediate-stage HCC. Mutations in cancer-related genes were detected through next-generation sequencing of resected tumor tissues and blood samples after resection. We identified five genes whose mutations in tissue samples were associated with survival outcomes, and confirmed that postoperative ctDNA (combined with AFP or not) could be used to stratify the recurrence risk and evaluate prognosis in patients with resected HCC.

## Method and Materials

### Patients and Samples

A total of 96 patients diagnosed with primary HCC were enrolled in this study. All patients provided written informed consent and received radical resection. The study was approved by the Ethics Committee of Xiangya Hospital Central South University (No. 201703377). After surgery, all patients were monitored regularly in the outpatient clinic with examinations of tumor markers, liver function and abdominal ultrasound every 3-6 months for two years, and then every 6 months. Further CT or magnetic resonance imaging scans were performed as needed if recurrence was suspected. Disease-free survival (DFS) was calculated from the date of liver resection to the date of diagnosis of tumor recurrence. Tumor burden was measured according to the modified Response Evaluation Criteria in Solid Tumors (mRECIST) (17). Tumor size was evaluated as the longest axial diameter of lesions.

Resected tumor tissue and peripheral blood samples (10 mL) within 7-10 days after surgery were collected from each patient. Peripheral blood was collected in Streck tubes (Streck, Omaha, NE, USA) and processed within 72 h to separate plasma and buffy coat (used to filter germline variants).

### DNA Extraction

Circulating free DNA (cfDNA) was isolated from plasma using a QIAamp Circulating Nucleic Acid Kit (Qiagen, Hilden, Germany). Buffy coat and tumor tissue DNA were extracted using the DNeasy Blood & Tissue Kit (Qiagen). DNA concentration was measured using a Qubit fluorometer and the Qubit dsDNA HS (High Sensitivity) Assay Kit (Invitrogen, Carlsbad, CA, USA). The size distribution of the cfDNA was assessed using an Agilent 2100 BioAnalyzer and a DNA HS kit (Agilent Technologies, Santa Clara, CA, USA).

### Targeted Capture Sequencing

Before library construction, 1 μg of tissue or buffy coat DNA was sheared to 300 bp fragments with a Covaris S2 Ultrasonicator (Covaris, Woburn, MA, USA). Indexed libraries were prepared from tissue, buffy coat and cfDNA using the KAPA Library Preparation Kit (Kapa Biosystems, Wilmington, MA, USA) as previously described (18). Libraries were then hybridized to custom-designed biotinylated oligonucleotide probes (Integrated DNA Technologies, Iowa, IA, USA). Capture probe for tissue samples was designed to cover whole coding regions or partial exons with mutations frequently detected (hot exons) of 1,021 genes (**Supplementary Table 1**). Capture probe for postoperative ctDNA samples was designed to cover coding sequences or hot exons of 293 genes, including frequently mutated genes in liver cancer or driver mutations in other cancer types (**Supplementary Table 1**). Matched tumor-normal sequencing was performed using Illumina 2×100 bp paired-end reads on an Illumina HiSeq 3000 instrument according to the manufacturer’s recommendations using a TruSeq PE Cluster Generation Kit v3 and a TruSeq SBS Kit v3 (Illumina, San Diego, CA, USA). Hybridization capture sequencing revealed a median value of the mean effective depth of coverage of 829× in resected tissue samples and 3470× in postoperative plasma samples (**Supplementary Table 2**).

### Sequencing Data Analysis

Terminal adaptor sequences and low-quality reads were removed from raw data of paired samples. Burrows-Wheeler Aligner (BWA, version 0.7.12-r1039) tool was used to align clean reads to the reference human genome (hg19). Somatic mutations were detected in tissue and ctDNA. Non-synonymous mutations including SNVs and InDels were detected using MuTect (version 1.1.4) and GATK, respectively, and hotspot variants were reviewed by NChot software. Mutations related to clonal hematopoiesis were filtered as previously described, including those in *DNMT3A, IDH1*, and *IDH2* and specific alterations within *ATM, GNAS* or *JAK2* (19, 20). The final candidate variants were all manually verified in the Integrative Genomics Viewer. Whether a tumor specific mutant was detected in the peripheral blood or not was defined as ctDNA (+) and ctDNA (-), respectively.

### Statistical Analysis

Nonparametric comparisons were preformed using Wilcoxon t test. Kaplan-Meier survival, univariate and multivariate Cox regression analyses were used to analyze associations between prognostic factors and survival. Univariate comparisons of proportion were analyzed using Fisher’s exact test. All statistical analyses were performed with SPSS (v.21.0; STATA, College Station, TX, USA) or GraphPad Prism (GraphPad Software 7.0, La Jolla, CA, USA) software. Statistical significance was defined as a two-sided P value of smaller than 0.05. LASSO analysis was used to identify prognostic features.

## Results

### Patient Characteristics

In this study, 96 patients with early- or intermediate-stage HCC were enrolled eligibly. Baseline characteristics were summarized in **Table 1**. All patients had pathologically confirmed primary HCC before surgical operation, with 78.1% (75/96) patients diagnosed as Barcelona Clinic Liver Cancer (BCLC) 0/A/B stage, and the remaining diagnosed as BCLC C stage. Enrolled patients including 85 males and 11 females. The median age at diagnosis was 50 years, ranging from 18 to 75 years. More than 95% (92/96) patients were infected with hepatitis B virus (HBV). The median diameter of the largest tumor was 5 cm, ranging from 1-18.5 cm. Microvascular invasion was seen in 50% (48/96) of cases, and multifocal lesions were occurred in 32.2% (31/96) of patients.

**Table 1:** Clinical characteristics of patients with primary hepatocellular carcinoma enrolled in this study.

Characteristics	Patients (n=96)
**Age at diagnosis, years**	
Median	50
Range	18-75
**Gender - no. (%)**	
Female	11 (11.5%)
Male	85 (88.5%)
**Maximum tumor diameter, mm**	
Median	5
Range	1-18.5
**Tumor morphology - no. (%)**	
Oligofocal	65 (67.7%)
Multifocal	31 (32.3%)
**BCLC Stage - no. (%)**	
0/A	60 (62.5%)
B	15 (15.6%)
C	21 (21.9%)
**HBV - no. (%)**	
Yes	92 (95.8%)
No	4 (4.2%)
**AFP, ng/ml**	
Median	126.6
Range	1.18-1473
>=400	40 (41.7%)
<400	56 (58.3%)
**CA199, U/ml^#^**	
Median	16.45
Range	0.06-162.5
>=27	28 (29.2%)
<27	56 (58.3%)
**Macrovescular Invasion - no. (%)**	
Yes	19 (19.8%)
No	64 (66.7%)
NA	13 (14.0%)
**MVI - no. (%)**	
M0	47 (49.0%)
M1	33 (34.4%)
M2	15 (15.6%)
NA	1 (1.0%)

AFP, alpha-fetoprotein; BCLC, Barcelona clinic liver cancer; CA-199, carbohydrate antigen 199; HBV, Hepatitis B virus; MVI, microvascular invasion; NA, not available.

^#^Twelve patients did not have baseline CA199 information (missing data).

### Mutational Profiling of Tumor Tissue Samples

**Figure 1:**
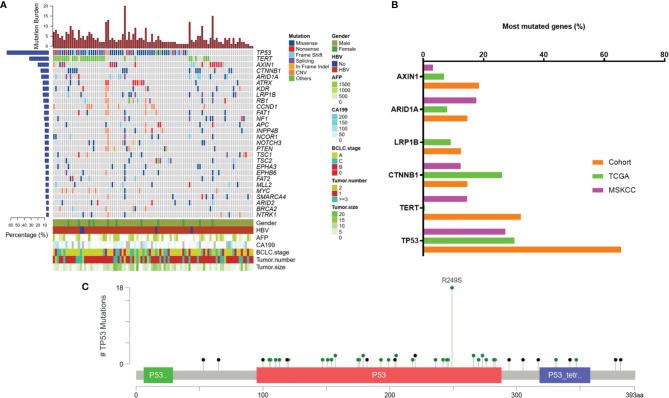
Mutation characteristics of tumor tissue samples. **(A)** Mutation profiles of tissue samples of 96 enrolled patients. **(B)** Frequently mutated genes in this cohort and the incidence of mutations in these genes in our cohort compared to that in public databases. **(C)** Lollipop diagram showing mutations in *TP53*. TCGA, The Cancer Genome Atlas; MSKCC, Memorial Sloan Kettering Cancer Center; AFP, alpha-fetoprotein; MVI, microvascular invasion; BCLC, Barcelona Clinic Liver Cancer; CA 19-9, carbohydrate antigen 19-9; HBV, hepatitis B virus.

Based on the targeted capture sequencing, a total of 1184 (including 292 CNVs) somatic mutations were detected in 96 tissue samples, with a positive detection rate of 100% (96/96, **Figure 1A** and **Supplementary Table 3**). The top three frequently mutated genes were *TP53* (65.6%, 63/96), *TERT* (32.3%, 31/96) and *AXIN1* (19.8%, 19/96) (**Figures 1A, B**). *TP53* mutations detected in 28.6% (18/63) of patients were concentrated at the R249S site, which was shown in **Figure 1C**. All the *TERT* mutations occurred in promoter regions (100%, 31/31). The mutational landscape of tumor tissue was further compared with data downloaded from Memorial Sloan Kettering Cancer Center (MSKCC) and the cancer genome atlas (TCGA) Cohort. As a result, the mutation frequencies of *TP53* (65.6% *vs* 27.2% and 30.3%, respectively), *AXIN1* (18.5% *vs* 3.2% and 6.9%, respectively), *TERT* (32.3% *vs* 14.5% and 0.6%, respectively) in our cohort were higher than that in MSKCC and TCGA database; while a relatively low detection rate of *CTNNB1* (14.6% *vs* 12.4% and 26.2%, respectively) was observed in our cohort, possibly implying the disparate molecular mechanisms of tumorigenesis between Eastern Asia population and Western or/and Caucasian population.

### Mutational Features of Tumor Tissue Related to Prognosis

**Figure 2:**
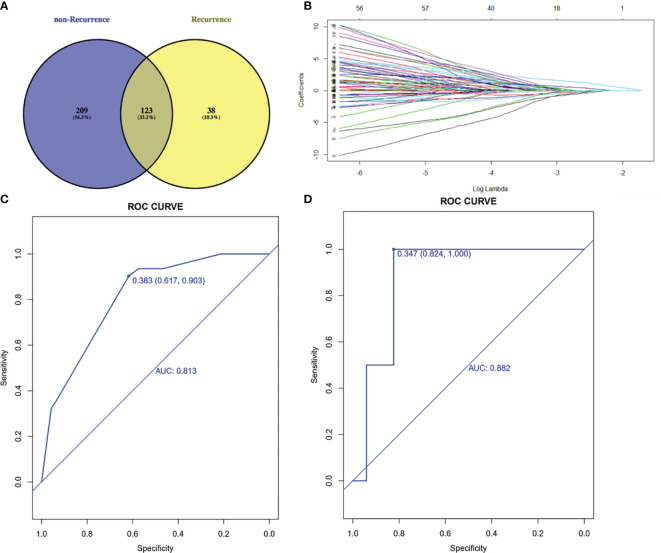
Lasso-Cox regression model. **(A)** 123 common genes mutated in both recurrence and non-recurrence group. **(B)** Least absolute shrinkage and selection operator (LASSO) regression model. **(C)** ROC curve of training cohort. **(D)** ROC curve of validation cohort. AUC, area under curve; ROC, receiver operating characteristic.

To select prognostic genetic features, we screened out 123 common genes in recurrence and non-recurrence group from 1021 cancer-related genes and performed the least absolute shrinkage and selection operator (LASSO) regression model on the basis of recurrence and DFS (**Figures 2A, B**). Five genetic features were identified in the training cohort: *AXIN1, CTNNB1, LRP1B, PDGFRA* and *TP53*. These five genes were also the frequently mutated genes in liver cancer. The area under the ROC curve was 0.813 and 0.882 in training and validation cohorts, respectively (**Figures 2C, D**).

**Figure 3:**
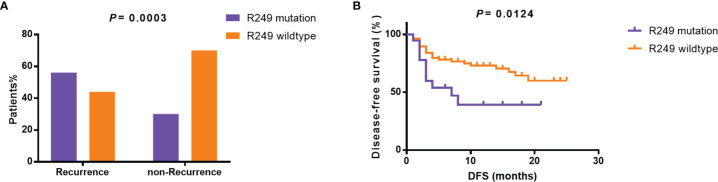
Relationship between *TP53* R249S mutation and prognosis. **(A)** Incidence of R249 mutation in the recurrent and non-recurrent groups. **(B)** Disease-free survival in the R249 mutation group and the R249 wildtype group. DFS, disease-free survival.

Furthermore, as shown in **Table 2**, specific gene mutations were related with patients’ clinical features. *TP53* mutations were associated with relapse (p=0.0106). *LRP1B* mutations were more likely to occur in older patients. The mutation rate in patients <50 years and ≥50 years was 2% and 21.6%, respectively (p=0.0042). Surprisingly, the difference in tumor size between *TERT*-wildtype and mutation group was also significant. The mutation rate was 23.1% in patients with tumor size ≥5 cm and 43.2% in patients with tumor size <5 cm (p=0.0358), which may suggest that *TERT* promoter mutations were more likely to be detected in tumor tissues in the early stage. *TP53* R249S mutation frequency was extremely high in liver cancer, which was considered to be closely associated with aflatoxin exposure and HBV infection (21, 22). In our cohort, *TP53* R249S mutation was detected in 18 patients and 94.4% (17/18) of them were identified with positive HBs-Ag. 55.5% (10/18) R249S mutation patients had recurred within 8 months, and 29.5% (23/78) patients without TP53 R249S mutation had recurred in 19 months (*P*=0.0003, **Supplementary Table 4** and **Figure 3A**). Patients with R249S point mutation showed a worse prognosis. The median DFS time was 7.0 months for R249S mutated group (n =18) and not reached for R249S wildtype group (n= 78), respectively (Log-rank test, *P* = 0.0124) (**Figure 3B**).

**Table 2:** The relationship between mutational status and clinical characteristics.

		TP53		*P*	TERT		*P*	AXIN1		*P*	PDGFRA		*P*	LRP1B		*P*
		M	W		M	W		M	W		M	W		M	W	
**Age**	<50	32	13	0.2877	12	33	0.2682	11	34	0.2825	1	43	0.1386	1	44	0.0042^**^
	>=50	31	20		19	32		8	43		5	47		11	40	
**Gender**	Male	54	31	0.2295	28	57	0.7052	17	68	0.8868	5	80	0.6791	10	75	0.5448
	Female	9	2		3	8		2	9		1	10		2	9	
**Tumor size**	>=5 cm	38	14	0.0947	12	40	0.0358^*^	11	41	0.7157	5	47	0.1386	8	44	0.3529
	< 5 cm	25	19		19	25		8	36		1	43		4	40	
	>= 3 cm	54	26	0.3871	22	58	0.0248^*^	16	64	0.9088	5	75	>0.9999	11	69	0.4076
	< 3 cm	9	7		9	7		3	13		1	15		1	15	
**BCLC stage**	0/A	39	21	0.358	22	38	0.4884	10	50	0.203	4	56	0.259	5	55	0.2799
	B	8	7		4	11		2	13		2	13		3	12	
	C	16	5		5	16		7	14		0	21		4	17	
**AFP**	< 200	33	18	0.785	20	31	0.2222	5	46	0.0059^**^	3	48	0.4207	5	46	0.6621
	>=200, < 400	4	1		2	3		0	5		1	4		1	4	
	>= 400	26	14		9	31		14	26		2	38		6	34	
**MVI**	Yes	36	12	0.044^*^	13	35	0.2438	12	36	0.2182	4	44	0.4139	7	41	0.7589
	No	26	21		18	29		7	40		2	45		5	42	
**Tumor number**	1	43	22	0.8857	22	43	0.1746	12	53	0.7348	4	61	0.5908	8	57	0.9888
	2	4	3		4	3		1	6		1	46		1	6	
	>= 3	16	8		5	19		6	18		1	23		3	21	
**HBV**	Yes	60	32	0.6868	28	64	0.062	18	74	0.7894	6	86	0.5978	11	81	0.44
	No	3	1		3	1		1	3		0	4		1	3	

Chi-square test was used and p < 0.05 was considered as significant. *, 0.01 ≤ p < 0.05; **, p < 0.01.

AFP, alpha-fetoprotein; BCLC, Barcelona Clinic Liver Cancer; HBV, hepatitis B virus; M, mutation; MVI, microvascular invasion; W, wildtype.

### ctDNA Positive Patients Had the Worse Prognosis

**Figure 4:**
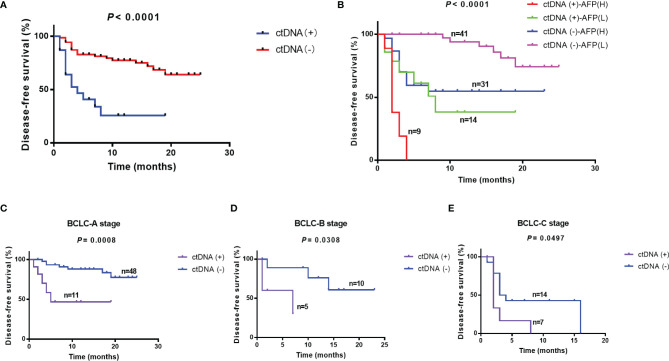
Relationship between ctDNA positivity and other clinical factors and prognosis. **(A)** Disease-free survival in ctDNA **(-)** and ctDNA (+) groups. **(B)** Prediction of prognosis using ctDNA combined with AFP. Prediction of prognosis using ctDNA in patients with different BCLC stages: **(C)** BCLC-A stage, **(D)** BCLC-B stage, **(E)** BCLC-C stage. AFP, alpha-fetoprotein; BCLC, Barcelona Clinic Liver Cancer.

Postoperative blood samples were collected within 7 days after surgery from all of the 96 patients. The sequencing results were classified based on whether mutations were detected in blood samples or not. Twenty-three patients were considered as ctDNA (+), and 60.9% (14/23) of them had recurrence; while 72 patients were ctDNA (-), and only 27.8% (20/72) patients had recurrence (P = 0.0059) (**Supplementary Figure 1**). The recurrence in 27 patients was in the liver only and 7 patients had distant metastases, and ctDNA positivity rate in these patients was 96.3% (26/27) and 100% (7/7), respectively. The median DFS time was 4.0 months in ctDNA (+) group, and the ctDNA (-) group had not reached the median time (Log-rank test, *P* < 0.0001) (**Figure 4A**). Multivariate Cox regression analysis showed that the postoperative ctDNA was an independent prognostic predictor of DFS (HR [hazard ratio]: 6.074, 95% Cl [confidence interval]: 2.648-13.929, P<0.001) and OS (HR: 4.829, 95% CI: 1.508-15.466, P=0.008 (**Table 3**).

**Table 3:** Univariate and Multivariate analysis of prognostic indicators.

	DFS				OS			
	Univariate analysis		Multivariate analysis		Univariate analysis		Multivariate analysis	
	OR (95% CI)	*P*	OR (95% CI)	*P*	OR (95% CI)	*P*	OR (95% CI)	*P*
Gender (female vs male)	1.999 (0.477-8.370)	0.343			0.804 (0.289-2.238)	0.804		
Age, years (<50 years vs ≥50 years)	0.738 (0.371-1.467)	0.386			0.782 (0.247-2.473)	0.676		
HBV (- vs +)	0.500 (0.151-1.649)	0.255			21.743 (0.000-2166292.226)	0.599		
CA199, U/mL (<40 U/mL vs ≥ 40 U/mL)	1.205 (0.543-2.674)	0.647			0.384 (0.050-2.977)	0.36		
**AFP, ng/mL (<400 ng/mL vs ≥400 ng/mL)**	3.363 (1.648-6.864)	0.001	3.919 (1.736-8.848)	0.001	8.365 (1.824-38.356)	0.006	6.696 (1.391-32.243)	0.018
**Tumor size (<5 cm vs ≥5 cm)**	4.361 (1.865-10.198)	0.001			4.929 (1.079-22.522)	0.04		
**MVI (M0 vs M1 &M2)**	5.405 (2.321-12.585)	<0.001	3.654 (1.453-9.187)	0.006	5.105 (1.119-23.300)	0.035		
**BCLC stage (stage 0 & A vs stage B &C)**	4.602 (2.222-9.532)	<0.001	1.974 (1.287-3.027)	0.002	6.425 (1.732-23.830)	0.005	2.033 (1.005-4.111)	0.048
**Tumor morphology (oligofocal vs multifocal)**	2.622 (1.321-5.203)	0.006			4.522 (1.359-15.044)	0.014	3.683 (1.061-12.783)	0.04
**Post-ctDNA (- vs +)**	4.551 (2.220-9.330)	<0.001	6.074 (2.648-13.929)	<0.001	7.011 (2.208-22.267)	0.001	4.829 (1.508-15.466)	0.008
**Macrovesular invasion (no vs yes)**	5.096 (2.404-10.798)	<0.001			7.222 (2.252-23.161)	0.001		

DFS, disease-free survival; OS, overall survival; OR, odds ratio; CI, confidence interval; HBV, hepatitis B virus; CA-199, carbohydrate antigen 199; AFP, alpha-fetoprotein; MVI, microvascular invasion; BCLC, Barcelona Clinic Liver Cancer; TMB, tumor mutation burden.

Factors in bold were included in multivariate analysis.

### Combined ctDNA With AFP and BCLC Stage Improved Prediction Performance

BCLC stage and baseline AFP level were indispensable clinical indicators to assess prognosis of liver cancer. In our study, BCLC stage and baseline AFP were independent prognostic predictors (**Table 3**). We classified patients based on postoperative ctDNA combined with baseline AFP or BCLC stage. The results showed that ctDNA combined with AFP would effectively predict the prognosis of patients after surgery. CtDNA (+)-AFP (H) (>= 400 ng/mL) patients had the worst prognosis and 77.8% (7/9) of them had relapsed; while ctDNA (-)-AFP (L) (<400 ng/mL) patients had the best prognosis, with less than 15% (6/41) had relapsed (Log-rank test, *P* < 0.0001). The median DFS time was 2.0, and 8.0 months in ctDNA (+)-AFP (H) (n=9) and ctDNA (+)-AFP (L) (n=14) groups, respectively; while ctDNA (-)-AFP (H) (n=31) and ctDNA (-)-AFP (L) groups (n=41) had not reached the median time statistically (Log-rank test, *P* < 0.0001) (**Figure 4B**).

In the previous study, BCLC stage was related to baseline ctDNA abundance. BCLC C patients had a higher ctDNA abundance than A and B stage patients (23). Combined ctDNA and BCLC stage may effectively predict the prognosis of patients after surgery. In BCLC A group, 11 patients were ctDNA positive and 48 patients were ctDNA negative, the ctDNA (-)-A patients had the better prognosis (P=0.0008) and the median DFS was 5 months in ctDNA(+)-A (n=11) and ctDNA(-)-A (n=48) had not reached the median DFS time. Similarly, the ctDNA (-)-B (n=10) and ctDNA (-)-C (n=14) patients had the better prognosis than ctDNA (+)-B(n=5) (P=0.0308) and ctDNA (+)-C (n=7) patients (P=0.0497). The median DFS was 7 months in ctDNA (+)-B and ctDNA (-)-B had not reach the median DFS time statistically. The median DFS was 2 and 3.5 months in ctDNA (+)-C and ctDNA (-)-C groups (**Figures 4C–E**).

## Discussion

The prognosis of patients with resectable hepatocellular carcinoma is affected by various factors. We portrayed the mutational spectrum of the surgical tumor tissues of HCC patients in our cohort, and finally determined 5 genes related to prognosis after resection through Lasso-Cox regression model. According to previous study, *TP53* is the most frequently mutated gene in HCC among all somatic mutated genes and its mutations were significantly associated with poorer overall survival in HCC patients (24). In various cancer types, *TP53* has a broad-spectrum mutation, but only in liver cancer, *TP53* mutations are highly concentrated at the R249 point, which was considered to be related to aflatoxin infection and positive HBs-Ag (21, 22). This is also observed in our research. Furthermore, we found that DFS and OS of patients harboring *TP53* R249 mutation were significantly shorter than those of wild-type patients. This finding may provide a clue for subsequent targeted treatment of liver cancer. Unexpectedly, TERT promoter mutations were related with smaller tumor sizes in our study. Previous studies revealed the inconsistent relationship between TERT mutations and tumor sizes. TERT promoter mutations in tissue samples did not correlated with tumor size (25–27). However, TERT mutations in ctDNA samples were correlated with large intrahepatic tumor size and increased mortality (28). One study compared the presence of TERT mutation in ctDNA and corresponding tumor tissue, and found that non-concordance (ctDNA-, tissue+) was associated with an early TNM stage. The TERT mutations were detected in 84.6% (11/13), 42.9% (3/7), 80% (4/5) and 55.6% (5/9) of patients with stage I, II, III and IV, respectively (27). These results were consistent with our findings to a certain degree.

In addition to the tumor mutational features and clinical factors, postoperative minimal residual disease (MRD) is considered as a high risk factor for tumor recurrence, which refers to postoperative tumor burden that cannot be identified with traditional diagnostic methods (29). In recent years, ctDNA-based sequencing has gradually been explored as a possible tool to identify MRD. Many previous studies demonstrated that detection of ctDNA after surgery was strongly associated with increased risk of disease recurrence (30–32). A few studies in HCC also revealed that the dynamic change of ctDNA after surgery could accurately and better evaluate patients’ prognosis and detect tumor occurrence prior to traditional strategies (13, 33). However, several studies generated conflicting data about the prognostic value of ctDNA (34). Indeed, low ctDNA concentration in early-stage cancers is a nonnegligible limitation of ctDNA analysis in early detection of relapse (35). Combination with other protein markers has been proved to improve the sensitivity and specificity of ctDNA for predicting prognosis (36, 37).

This prospective study evaluated the clinical utility of ctDNA for detection of MRD in HCC. We found that ctDNA positivity in the immediate post-operative period was significantly associated with worse DFS and OS independently, suggesting the clinical significance of ctDNA-based MRD detection. We further combined ctDNA with clinical indicator AFP and BCLC stage and found that patients with positive postoperative ctDNA-AFP (H) subgroups and patients with positive ctDNA and BCLC staging C subgroups had the worst prognosis. It is not enough for us to evaluate the possibility of postoperative recurrence by relying solely on traditional clinical indicators. Especially for patients with earlier stage tumors, postoperative ctDNA has unique advantages in identifying high-risk recurrence patients.

In summary, postoperative ctDNA detection has great potential value in clinic, and patients with positive ctDNA after surgery should receive individualized medication to improve the survival time.

## Data Availability Statement

The original contributions presented in the study are publicly available. This data can be found here: https://ngdc.cncb.ac.cn/gsa-human, HRA001983.

## Ethics Statement

The studies involving human participants were reviewed and approved by Ethics Committee of Xiangya Hospital Central South University. The patients/participants provided their written informed consent to participate in this study.

## Author Contributions

KY and LZ contributed to the conceptualization and design. KY, QF, MY, TF and ZL contributed to the data curation and writing. DW, LX, and GL analyzed and interpreted the data. RC contributed to the conceptualization. All authors contributed to the article and approved the submitted version.

## Conflict of Interest

MY and RC are employees of Geneplus-Beijing.

The remaining authors declare that the research was conducted in the absence of any commercial or financial relationships that could be construed as a potential conflict of interest.

## Publisher’s Note

All claims expressed in this article are solely those of the authors and do not necessarily represent those of their affiliated organizations, or those of the publisher, the editors and the reviewers. Any product that may be evaluated in this article, or claim that may be made by its manufacturer, is not guaranteed or endorsed by the publisher.

## Supplementary Material

Supplementary Figure 1 The proportion of ctDNA positivity in the recurrence and non-recurrence groups.
