# Supplementary figures and images for: Prognostic Value of Postoperative Circulating Tumor DNA in Patients With Early- and Intermediate-Stage Hepatocellular Carcinoma
Source: Front Oncol. 2022 Mar 4;12:834992. doi: 10.3389/fonc.2022.834992 (PMC8931326; doi:10.3389/fonc.2022.834992)

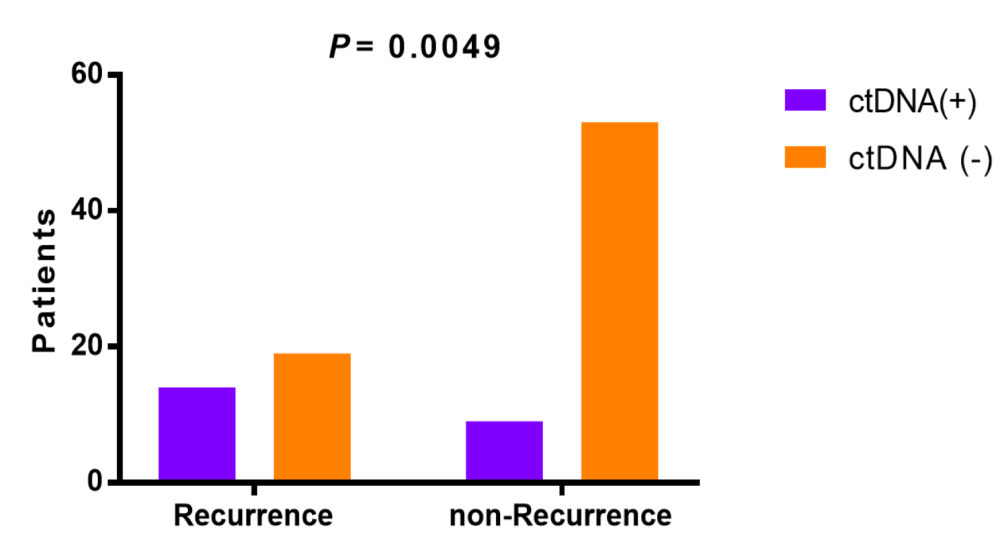

Supplement: Supplementary Figure 1 — The proportion of ctDNA positivity in the recurrence and non-recurrence groups. [file Image_1.tiff]
